# Genome Sequence of a Microvirus Recovered from Wastewater in Arizona, USA, in October 2020, Encodes a Previously Undescribed DNA-Binding Protein

**DOI:** 10.1128/mra.00337-22

**Published:** 2022-08-31

**Authors:** Abriana Smith, Nicole Kaiser, Allan Yanez, Tyler Perleberg, Amir Elyaderani, Peter Skidmore, Sangeet Adhikari, Erin M. Driver, Rolf U. Halden, Arvind Varsani, Matthew Scotch, Temitope O. C. Faleye

**Affiliations:** a College of Health Solutions, Arizona State University, Tempe, Arizona, USA; b Biodesign Center for Environmental Health Engineering, Biodesign Institute, Arizona State University, Tempe, Arizona, USA; c School of Sustainable Engineering and the Built Environment, Arizona State University, Tempe, Arizona, USA; d OneWaterOneHealth, Arizona State University Foundation, Tempe, Arizona, USA; e Biodesign Center for Fundamental and Applied Microbiomics, Center for Evolution and Medicine, School of Life Sciences, Arizona State University, Tempe, Arizona, USA; Queens College CUNY

## Abstract

We describe the genome of Microvirus-AZ-2020, which was identified from wastewater in Arizona, USA, in October 2020. Microvirus-AZ-2020 belongs to subfamily *Gokushovirinae* and contains six (five known and one hypothetical) open reading frames (ORFs), each with >40 codons. HHPred analysis and Colabfold structure prediction suggest that the hypothetical ORF encodes a previously undescribed putative DNA-binding protein.

## ANNOUNCEMENT

Microviruses are bacteriophages in the *Microviridae* family and are circular single-stranded DNA viruses with icosahedral capsids. Metagenomic studies have identified microviruses in a range of environments (1, 2). We describe the genome of Microvirus-AZ-2020, which was identified from wastewater collected in Arizona, USA, in October 2020.

**FIG 1:**
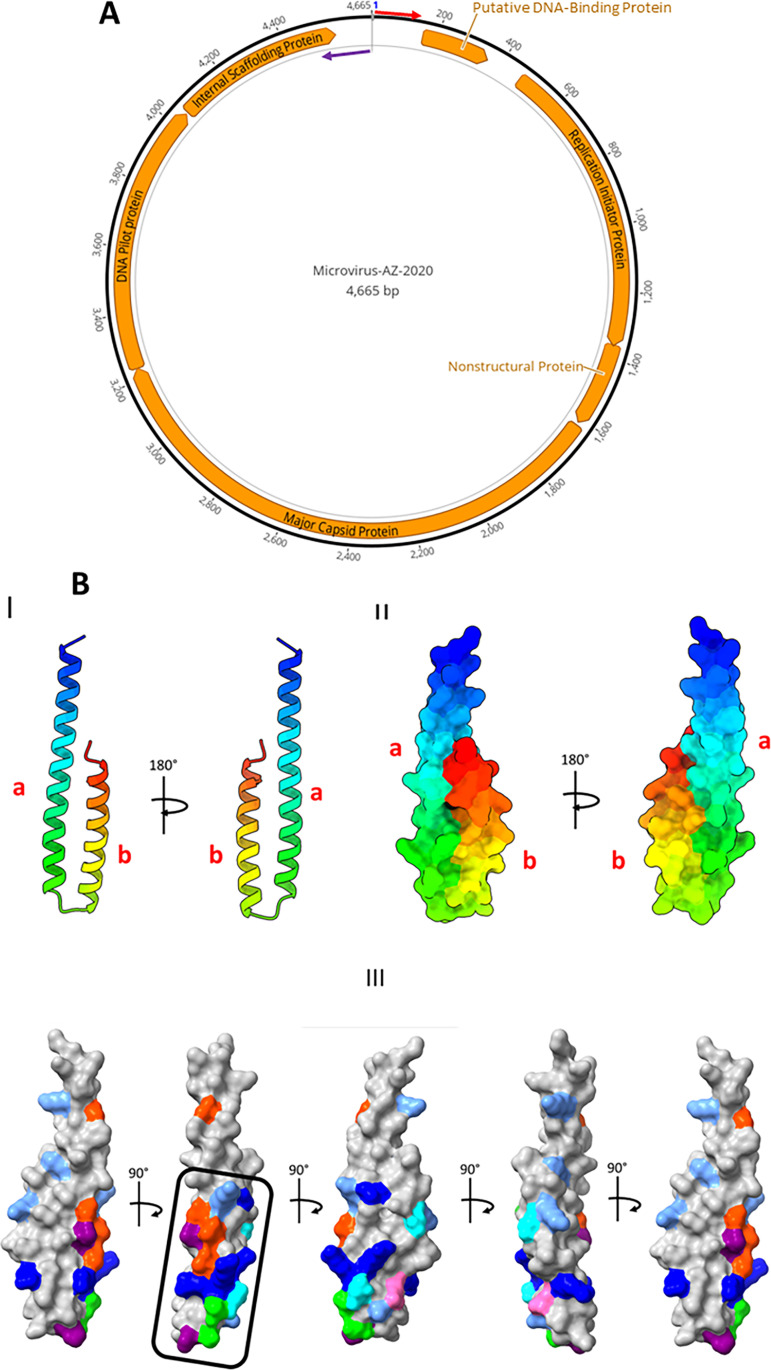
(A) Genome map of Microvirus-AZ-2020. The binding site of the primers used to amplify the complete genome is indicated by colored arrows. (B) Colabfold-predicted structure of the putative protein in panel A. The structure was viewed and annotated in ChimeraX. I is shown in ribbon view, while II and III are shown in surface view. I and II show the amino terminus to the carboxyl terminus from blue to red. In I and II, a and b refer to the two alpha-helices from amino to carboxyl terminal, respectively. III shows the result of DNA-binding residue prediction layered on the predicted three-dimensional structure of the molecule (blue, cornflower blue, purple, pink, cyan, green, and orange indicate amino acid residues R, K, H, Q, N, S, and T, respectively). The region highlighted with a black box in III shows clustering of some predicted DNA-binding residues.

The 2-L postfiltration sample (450 nM) was concentrated to 2 mL by ultrafiltration using a 10,000-molecular-weight-cutoff centrifugal filter. The concentrate was subjected to nucleic acid extraction (QIAamp minikit), pan-enterovirus reverse transcription (RT)-PCR (3), library preparation (KAPA HyperPlus library kit), and paired-end (2 × 250-bp) sequencing (MiSeq system; Illumina). Raw reads were trimmed and *de novo* assembled using Trimmomatic v0.36 and metaSPAdes v3.15.3, respectively, on the KBase platform (4). Contigs were identified using a BLASTn search of the GenBank database (5). The proportion of reads mapped to Microvirus-AZ-2020 (template) and the depth of coverage were determined using Bowtie2 v2.3.2 (3). The Microvirus-AZ-2020 open reading frames (ORFs) were predicted using Prokka v1.0.0 (6) and DNA Master v5.23.6 (7). Functional annotation of the predicted ORFs was performed via a BLASTp search of the nonredundant protein sequence database using the BLAST-All-Genes option in DNA Master. Unannotated ORFs were subjected to HHPred analysis (8, 9) and protein structure prediction using ColabFold (10), which combines the fast homology search of MMseqs2 with AlphaFold2. DNA-binding residues were predicted using the DRNApred server (11). All software was used with default parameters unless otherwise specified. Primers MazF (5′-GTGGCGAAGCCGGCATGGGTTGTTAGGG-3′) and MazR (5′-GTGGCGAAGCCGGCATGGGTTGTTAGGGAGAAACCC-3′) (Fig. 1A) were used to confirm the presence of virus in the sample by PCR using Phusion green master mix with the following reaction conditions: 94°C for 3 min, 40 cycles of 94°C for 30 s, 55°C for 30 s, and 68°C for 6 min, and finally 68°C for 10 min.

Microvirus-AZ-2020 (4,665 nucleotides [nt] [GC content, 51%]) was *de novo* assembled from 2,561 reads (0.07% of the 3,913,700 trimmed reads [depth of coverage, 111×]), circularized via terminal redundancy, and determined by BLASTn to be most similar to Apis mellifera-associated microvirus (subfamily *Gokushovirinae*) (GenBank accession MH992184 [12]) (query coverage, 77%; pairwise identity, 67.75%; E score, 0.0). Both Prokka (Fig. 1A) and DNA Master (Table 1) predicted six ORFs with >40 codons. A BLASTp search of the nonredundant protein sequence database annotated five of the predicted ORFs as encoding replication initiator protein, nonstructural protein, major capsid protein, DNA pilot protein, and internal scaffolding protein (Table 1 and Fig. 1A). No function could be assigned to the sixth (hypothetical) ORF using BLASTp. However, 8 of its top 10 HHPred analysis hits were transcription regulation proteins (probability, 67.2% to 83.35%). ColabFold structure prediction showed that the protein has a helix-turn-helix motif (Fig. 1BI), suggesting that it is a potential DNA-binding protein. When it was layered on the three-dimensional structure, DRNApred predicted that the DNA-binding residues spanned both α-helices but clustered toward the lower half (from the amino end) of helix a (Fig. 1BIII).

**TABLE 1:** Details of predicted ORFs in Microvirus-AZ-2020

ORF no.	Nucleotide positions of ORF	Length (nt)	Length (amino acids)	Start codon	Stop codon	GenBank accession no. of most similar protein sequence (species)	Alignment (%)	Identity (%)	BLASTp-predicted function
1	152–358	207	68	ATG	TGA	AZL82829 (Apis mellifera-associated microvirus 13)	100	31.34	Hypothetical protein
2	468–1361	894	297	GTG	TGA	AXH74636 (*Microviridae* sp.)	100	61.74	Replication initiator protein
3	1358–1612	255	84	ATG	TGA	AZL82825 (Apis mellifera-associated microvirus 13)	100	54.32	Nonstructural protein
4	1621–3234	1614	537	ATG	TGA	AZL82828 (Apis mellifera-associated microvirus 13)	100	70.30	Major capsid protein
5	3237–4046	810	269	ATG	TGA	AZL82826 (Apis mellifera-associated microvirus 13)	92.2	50.96	DNA pilot protein
6	4051–4557	507	168	ATG	TAA	AZL82827 (Apis mellifera-associated microvirus 13)	94.9	57.05	Internal scaffolding protein

We describe the genome of Microvirus-AZ-2020, a microvirus recovered from wastewater in Arizona, USA, in October 2020 that encodes a previously undescribed putative DNA-binding protein. Surveillance of microviruses is needed to improve our understanding of their diversity and unexplored protein repertoire.

### Data availability.

The mapped reads and microvirus genome described in this study have been deposited in the SRA and GenBank under accession numbers SRR18497324 and ON111452, respectively.
